# Whole-genome sequencing and bioinformatic tools powered by machine learning to identify antibiotic-resistant genes and virulence factors in Escherichia coli from sepsis

**DOI:** 10.1099/mgen.0.001465

**Published:** 2025-08-11

**Authors:** Nishitha R. Kumar, Tejashree A. Balraj, Kerry K. Cooper, Akila Prashant

**Affiliations:** 1 Department of Biochemistry, JSS Medical College and Hospital, JSS Academy of Higher Education and Research, Mysuru, Karnataka, India; 2 Department of Microbiology, JSS Medical College and Hospital, JSS Academy of Higher Education and Research, Mysuru, Karnataka, India; 3 School of Animal and Comparative Biomedical Sciences, University of Arizona, Tucson, AZ, USA; 4 BIO5 Institute, University of Arizona, Tucson, AZ, USA; 5 Department of Medical Genetics, JSS Medical College and Hospital, JSS Academy of Higher Education and Research, Mysuru, Karnataka, India

**Keywords:** community acquired infections, *Escherichia coli*, hospital acquired infections, multidrug resistance, multilocus sequence typing, phylogentic analysis, sepsis, supervised machine learning, virulence factors, whole-genome sequencing

## Abstract

Extended-spectrum *β*-lactamase-producing *Escherichia coli* poses a global public health threat. Here, we performed a hospital-based study that reinforced the necessity for rapid antimicrobial resistance (AMR) and virulence gene mapping of clinical *E. coli* isolates. Whole-genome sequencing of 18 sepsis-causing *E. coli* strains was performed to identify multidrug resistance (MDR) and virulence factor genes and to correlate these with antibiotic use in patients with sepsis. We identified various global and emerging MDR sequence types, utilizing a supervised machine learning approach to elucidate the relationship between genome content and AMR profiles across 17 antimicrobial classes, ensuring unbiased analysis. Known AMR genes were correlated with resistance phenotypes, and several crucial and novel AMR genes were identified. The feature selection methodology involved processing the genome into overlapping 13 bp k-mer features using a two-step selection process. Logistic regression with nested cross-validation and synthetic minority oversampling technique confirmed the robustness of the model. The combination of Machine Learning (ML) algorithms facilitates the discovery of nonlinear interactions and complex patterns within genomic data, which may not be readily apparent using conventional genomic analysis alone. This will enable the identification of novel biomarkers and genetic determinants of AMR profiles. The integration of genomic data with ML models can be used to quickly predict AMR, allowing for more targeted and personalized treatment strategies that are not typically achieved by traditional AMR surveillance methods. Our findings tailor the research approaches for patients with sepsis, particularly with AMR *E. coli*, highlighting the importance of prompt surveillance, robust infection control, optimized antibiotic stewardship and integrated genomic and epidemiological analysis to control MDR bacteria transmission, ultimately improving patient outcomes and safeguarding public health.

**Impact Statement**

Sepsis is a major global health concern. Septic shock is a subclass of sepsis distinguished by metabolic, cellular and circulatory defects that increase mortality risk among patients with sepsis. Owing to their increased prevalence and pathobiological, molecular, genomic and medical complications, sepsis and septic shock pose a growing worldwide burden and a formidable challenge for clinicians. Reports have revealed that India is among the highest antibiotic consumers worldwide, and broad-spectrum empiric antibiotic therapy collectively poses a substantial healthcare burden in the intensive care unit settings, which is responsible for lengthy hospital stays, high hospital-associated costs and high mortality. Multidrug-resistant (MDR) bacterial infections cause ~700,000 deaths annually, with the possibility of this number increasing to more than 10 million by 2050. Implementing strategies for controlling and preventing illness, including prudent antibiotic stewardship, strict adherence to hand hygiene protocols, comprehensive environmental disinfection regimens and timely detection of MDR micro-organisms, is critical for restraining the transmission of antimicrobial resistance pathogens. Early diagnosis and prompt initiation of treatment are essential for enhancing clinical outcomes and reducing the mortality rate of sepsis.

## Data Summary

1. Supplementary files have been uploaded on the Microbiology Society’s Figshare account: https://doi.org/10.6084/m9.figshare.27204585.v1[1].

2. Genome data were submitted to the National Center for Biotechnology Information (NCBI) under the BioProject database ‘PRJNA821629’, with accession numbers.

## Introduction

Extended-spectrum *β*-lactamase (ESBL)-producing *Escherichia coli* is a major public health concern, substantially increasing morbidity and mortality worldwide. In 2017, the World Health Organization (WHO) [2] designated ESBL-producing *Enterobacterales*, including *E. coli*, as high-risk pathogens for research and antibiotic policy. *E. coli* infections persist in severe and potentially life-threatening medical conditions, such as sepsis, owing to antibiotic resistance and virulence factors (VFs) [3]. *E. coli* clinical isolates display resistance to various antibiotic drug classes, including (a) aminoglycosides, amikacin [4], gentamicin [5] and tobramycin [6]; (b) carbapenems: imipenem [7,8], meropenem [8,9], doripenem [10] and ertapenem [8]; (c) extended-spectrum beta-lactam: cephalosporins [11]; (d) fluoroquinolones: ciprofloxacin [12] and levofloxacin [13]; and (e) piperacillin group: piperacillin/tazobactam [14], indicating a high prevalence of multiple antibiotic resistance in clinical *E. coli*. Plasmid-mediated enzymes hydrolyse the *β*-lactam ring compounds, conferring resistance to ESBLs or AmpC-*β*-lactamases [15]. These ESBLs and carbapenemases belong to different molecular classes (A–D) and exhibit variations in *β*-lactamase genes, including AmpC-*β*-lactamases (*ACT*), sulfhydryl variable (*SHV*), temoneria (*TEM*) and cefotaximase-munich (*CTX-M*) [16]. Moreover, ESBLs have been found to confer resistance to multiple generations of cephalosporins, posing a substantial challenge for treatment. During sepsis, the immune response to intracellular *E. coli* involves the recognition of pathogen-associated molecular patterns on the surface receptors for *E. coli*. This recognition is facilitated by pattern recognition receptors, specifically toll-like receptors [17]. Consequently, an inflammatory response is triggered and mediated by neutrophils and macrophages [18] that engulf bacteria and prevent their replication. However, * E. coli* strains with antimicrobial resistance (AMR) genes have evolved strategies to evade the host immune response, mainly targeting immune cells by disrupting the function of the antigen-presenting cells [19]. These resistant strains also disrupt signalling molecules that are crucial for microbial invasion. Antibiotic resistance in *E. coli* sepsis poses a substantial challenge during hospitalization, often necessitating the extensive use of broad-spectrum antimicrobials. However, *E. coli* resistance to multiple antimicrobials in the clinical settings implies a persistent and worsening infection, even after broad-spectrum antibiotic treatment [20]. These mechanisms complicate the management and control of intracellular *E. coli* infections. Globally, sepsis is a substantial health issue with high mortality rates, especially in low- and middle-income countries such as India, which currently lack effective control and management of sepsis [21,22]. The emergence of AMR *E. coli* is caused by the extensive and improper usage of antibiotics in both healthcare and community settings. Therefore, understanding these evasion strategies can help create novel scientific methods to prevent and treat *E. coli* infections, particularly in patients with sepsis.

Recently, reports on bioinformatic applications have exponentially increased. Bioinformatics plays a crucial role in advancing the identification of AMR profiles of pathogenic micro-organisms with high accuracy and efficiency [23]. Computational tools facilitate the detailed understanding of the resistance mechanisms found in pathogenic bacteria and are highly accessible to clinicians for treatment regimens [24]. For example, Luo *et al*. used bioinformatic analysis to create a predictive model that could recognize active latent tuberculosis with 88% sensitivity and 91% specificity [25]. Novel microbial genomic techniques, particularly whole-genome sequencing (WGS), have numerous advantages over conventional molecular techniques [26]. Similarly, microbial genomic data play a crucial role in machine learning-based AMR gene discovery. Integrating WGS with bioinformatic tools and machine learning enhances the identification of AMR and VFs, considerably advancing knowledge and effectively managing sepsis and septic shock caused by *E. coli*. By leveraging genomic sequences, the study protocol included various machine learning algorithms to predict and characterize AMR genes. These k-mers are fed into supervised machine learning algorithms as matrices of frequencies or counts to identify the features that best distinguish each category for accurate predictions. Identifying AMR and VF genes from microbial genomic data of patients with sepsis is crucial for controlling the dissemination of AMR pathogens [27]. Furthermore, it helps to optimize antibiotic administration and appropriate treatment choices, ultimately improving patient outcomes. This study aimed to utilize the transformative potential of WGS to identify the AMR and VF genes within clinically isolated *E. coli* strains, emphasizing the critical significance of these insights for advancing our understanding of *E. coli* infections and guiding more precise and effective strategies for diagnosis, treatment and infection control.

## Methods

### Patient inclusion criteria

A uniform inclusion criterion for patients with acute organ dysfunction represented by an increase of at least two or more points on the Sequential Organ Failure Assessment (SOFA) was established. Patients meeting four or more of the following criteria for prognosis and diagnosis of sepsis were included in this study: an increase in quick SOFA by <20 breaths/min, ≤100 mmHg blood pressure and >90 beats/min; white blood cell count of >12,000/mm^3^ or <4,000/mm^3^; >38.3 °C (101 °F) fever; hypothermia (<36 °C core temperature, <96.8 °F); significant oedema or positive fluid balance >20 ml kg^−1^ over 24 h; hyperglycaemia (>140 mg dl^−1^ with type 2 diabetes); elevated C-reactive protein (<5 mg l^−1^) and procalcitonin in serum (0.1–0.5 nmg ml^−1^) according to JSS lab cut-off values; arterial hypoxaemia partial pressure of paO2/FiO2<300; an acute drop in urine output <0.5 ml kg^−1^ h^−1^ for at least 2 h despite fluid resuscitation or ~35 ml h^−1^ for a 70 kg person; creatinine >0.5 mg dl^−1^; International Normalized Ratio >1.5 or activated partial thromboplastin time >60; absent bowel sounds with ileus; platelet count <100,000; and total bilirubin >4 mg dl^−1^. Only 20 selected isolates were left for further analysis after applying the inclusion criteria.

### Sample collection and bacterial extraction

Blood cultures from 87 patients were collected from various intensive care units (ICUs), including medical, respiratory and surgical ICUs, at JSS tertiary care hospital in Southern India. These cultures were processed for bacterial identification and antibiotic susceptibility testing (AST) using the bioMerieux BACT/ALERT system (an automated system that detects bacterial growth through CO_2_ production). Samples showing no growth after 72 h were considered insignificant, whereas buoyant bacterial culture suspensions were isolated from MacConkey agar media under aseptic conditions. MacConkey agar was used to isolate the bacteria due to its selective properties including bile salts and crystal violet which inhibit the growth of Gram-positive bacteria or growth of other bacterial contamination. Pure isolates of Gram-negative *E. coli* underwent phenotypic identification and AST. The isolated strains were suspended in 3.0 ml of sterile solution (0.4–50% NaCl; pH 4.5–7.0) in clear plastic test tubes, adjusted to 0.5 McFarland turbidity using a DensiCheck instrument, loaded into VITEK 2 compact [28] and incubated at 35.5±10 °C [29]. At 15-min intervals, the colourimetric reagent containing the card was automatically transported to an optical system for data collection. As indicated by signalling flags, positive reactions were collected aseptically after incubation and subcultured on MacConkey agar at 37 °C for 18–24 h. Isolated colonies were subjected to Gram staining. Growth suspensions were prepared by inoculating the isolated colonies in 3.7% Todd Hewitt broth (Thermo Fisher Scientific) and incubating at 80 r.p.m. for 4 h to obtain bacteria in the log phase.

### Subculturing and bacterial purification

Twenty clinical bacterial isolates obtained from patients with sepsis were identified as *E. coli*. They underwent AST against 16 antimicrobials using the Kirby–Bauer disc diffusion method following the guidelines of the Clinical Laboratory Standard Institute [30]. Various antibiotic discs were used to determine the susceptibility of *E. coli* isolates (Table 1). Isolates exhibiting resistance to more than two antimicrobial classes were classified as multidrug-resistant (MDR) bacteria. The ESBL phenotype was determined using the double-disc synergy method on Mueller–Hinton agar plates by placing ceftriaxone and ceftazidime discs 20 mm apart from the amoxicillin and clavulanic acid discs. An apparent inhibition zone around the ceftazidime or ceftriaxone discs near the clavulanic acid-containing disc confirmed ESBL production. Colonies obtained from these plates were examined for Gram staining characteristics. Gram staining was performed as the confirmatory step. This double-check is necessary under aerobic conditions as some Gram-positive bacteria may occasionally grow on MacConkey agar, leading to potential misidentifications. Gram staining allows for direct visualization of the cell wall structure, providing an additional layer of validation to ensure the accuracy of the isolation process. This step is crucial for maintaining the reliability and specificity of our findings, as the accurate identification of Gram-negative *E. coli* is required for further genomic DNA (gDNA) extraction to proceed with WGS. Growth suspensions were inoculated by emulsifying AMR *E. coli* colonies in 3.7% Todd Hewitt broth and incubating at 80 r.p.m. for 4 h to obtain bacteria in the log phase. The bacterial biomass was cryopreserved in 50% v/v glycerol at −80 °C for future use.

**Table 1.** Number of phenotypic data of resistance and susceptibility in 18 *E. coli* isolates. No statistical difference was found between resistance and sensitivity (chi-square *P*-value <0.001) either overall or by antimicrobial grouping

Sl no.	Antibiotic	Abbreviation	Resistant	Sensitive
1	Amikacin (30 mg)	AMK	6	12
2	Amoxicillin/clavulanic acid (20/10 mg)	AMC	12	6
3	Ampicillin (10 mg)	AMP	16	2
4	Cefepime (30 mg)	FEP	14	4
5	Cefoperazone/sulbactam (30 mg)	CZO	11	7
6	Ceftriaxone (30 mg)	CRO	16	2
7	Cefuroxime (30 mg)	CXM	16	2
8	Cefuroxime axetil (30 mg)	COL	16	2
9	Ciprofloxacin (5 mg)	CIP	16	2
10	Gentamicin (10 mg)	GEN	13	5
11	Imipenem (10 mg)	IMP	9	9
12	Meropenem (10 mg)	MEM	7	11
13	Nalidixic acid (30 mg)	NAL	16	2
14	Piperacillin/tazobactam (25 mg)	PIP	9	9
15	Trimethoprim/sulfamethoxazole (25 mg)	SXT	11	7
16	Tigecycline (32 µg)	TGC	3	15

### DNA extraction and WGS

gDNA of 20 *E. coli* strains from patients with sepsis was isolated using the Qiagen QIAamp DNA Mini Kit (QIAGEN) [31], as shown in Table S1 (available in the online Supplementary Material). The DNA of the 20 isolates was sent for WGS to MedGenome (Bengaluru, India). Before proceeding to sequencing, the 20 samples were quantified using Qubit High Sensitivity D1000 DNA screen tapes (Agilent, Cat#5067–5584) and Qubit™ dsDNA Quantification Assay Kits (Thermo Fisher Scientific) in MedGenome. DNA purity was measured using the QIAxpert system, and the DNA integrity was assessed using 0.8% agarose gel electrophoresis (Fig. S1). Illumina sequencing libraries were constructed for each isolate, and 150 bp Pair End (PE) reads were generated using an Illumina HiSeq instrument, yielding 3.5–7.0 million PE reads/sample.

### Genome assembly and annotation

For bioinformatic analysis, quality control of 20 genome pair read sequences was performed using FastQC v10. Phred scores above >30 indicated high-quality bases. During data cleaning, reads that exhibited specific characteristics, such as those containing over 40% low-quality bases, were excluded to ensure the reliability of subsequent analyses. In addition, reads with more than 10% unidentified bases (N) and adapter sequences were excluded. Whole-genome high-throughput sequencing yielded 154 high-quality reads from 18 *E. coli* genomes. However, these two bacterial genomes did not meet the quality criteria and were thus excluded from further analysis. Raw genome data were submitted to the National Center for Biotechnology Information (NCBI). Genome assembly and annotation of 18 *E. coli* genomes were performed using genome *E. coli* K-12 strain MG1655 as the reference genome (accession NC_000913.3). This reference genome was used to annotate the 18 *E. coli* strains which was driven by various factors, such as *E.* *coli* K-12 MG1655, which is regarded as the standard reference for *E. coli* due to its widely used reference-based genome studies worldwide with extensive characterization for comparative genomic studies. *E.* *coli* K-12 MG1655 served as a reliable baseline for the identification of conserved genes and core functionalities across *E. coli* strains. This approach of comparing the genomes of *E. coli* K-12 MG1655 was used to maintain the uniformity of the genetic elements. This extensively studied reference genome ensures high accuracy and comparability with previously published research. To address the potential limitations of the *de novo* assembly and compare our 18 *E. coli* genomes with *E. coli* K-12 MG1655 as a reference, bioinformatic tools were used to detect homologous sequences and regions of divergence. This phased approach provided depth and breadth for understanding the pathogenicity of *E. coli* genomes.

*E. coli* genomes were clustered by similarity and identity based on >99.9% similarity, as shown in Fig. S2. Furthermore, the *de novo* assembled genome was scaffolded and rearranged using the reference genome, *E. coli* K-12 strain using Ragout, and gap-filled using FGAP software, resulting in a single scaffold as shown in Busco assessment results in Fig. S3. During this process, the Ragout filtered out the contaminated scaffolds as well. Furthermore, the single Ragout master single scaffold was considered as the final assembly.

### Pathogenicity island

Various genomic elements were analysed, including genomic islands, repeat regions, transfer elements, plasmids and insertion sequences. Repetitive sequences were predicted using the Repeat Masker tool [32], and tandem repeats were scrutinized using the Tandem Repeat Finder (TRF) tool [33]. Gene annotation was performed using blastp searches (*E*-value <1, minimum alignment length percentage of 40%) to determine the functional significance of the genetic content. The re-annotation of genetic elements using blastp was performed despite having annotated the strains using the reference genome NC_000913.3, which was necessary to address specific gaps and ensure a comprehensive characterization of strain-specific and unique genes. The initial annotation with the reference genome provided a strong foundation for conserved genes. Still, re-annotation with blastp allowed us to identify and characterize genes that may not be present in the reference genome or exhibit significant sequence divergence. An *E*-value threshold of <1 may appear lenient as an initial filter for capturing a broad range of potential matches, particularly for divergent sequences that still hold biological relevance. However, the *E*-value was not the sole criterion; additional layers of stringency, such as percentage identity and alignment coverage, were applied to refine the results. For functional annotation, a minimum identity percentage of 70% was used as the primary criterion. This threshold balances the sensitivity and specificity, ensuring the inclusion of homologous sequences while minimizing false positives. The additional criterion of a minimum alignment length of 40% of the query sequence ensured the biological significance of the matches, particularly for partial or fragmented genes. The annotation was performed using different tools, including blastp and others, each with its default parameters. We maintained the default parameters for the tools optimized for specific tasks. These settings were validated for reliability by their developers. However, when blastp was used for re-annotation, the stated parameters (*E*-value <1, minimum alignment length of 40% and percentage identity of 70%) were applied to standardize the analysis. The investigation involved the comparison of four databases in a standalone environment. The data are presented in Table S2.

The tools below were used to analyse functional annotations or metabolic pathway predictions. This analysis confirmed that employing multiple databases ensures comprehensive and cross-validated datasets. However, we acknowledge the variability in parameters across databases and have adapted our interpretation accordingly, relying on stringent criteria such as percentage identity and alignment length for blast-based annotations to ensure consistency.

The following databases were used to identify pathogenicity islands in the *E. coli* genome. (1) Kyoto Encyclopedia of Genes and Genomes (KEGG) v4 functions were assigned through the KAAS annotation server [34] (this database uncovers metabolic pathway and associated functions of genes); (2) Clusters of Orthologous Groups (COG) v12 annotation clusters were assigned using the COG classifier Python programming [34,35] (this website enables the classification of genes into orthologous groups for evolutionary and functional analysis; (3) Gene Ontology (GO) v10 panther [36] database powered gene ontology for biological processes, molecular functions and cellular components by providing structural terms to characterize gene functions (this database enhances our understanding of the roles that genes play within biological processes); (4) noncoding RNA (ncRNA) databases tRNA: tRNAscan-SE v1.3.1 [37], rRNA: RNAmmer in Prokka [38] v1.2 and sRNA: Rfam database v8 [39]: subdivided into tRNA, rRNA and sRNA (this resource identifies ncRNA molecules, including transfer RNA ‘tRNA’, ribosomalRNAs ‘rRNA’ and smallRNAs ‘sRNA’, respectively). Circos [40] was used to visualize the functional annotation in a circular form to provide a comprehensive view of metabolism and genetics.

### *In silico* subtyping identification

Sequence types (STs) were identified through MLST, mapping the sequences to the PubMLST (*E*. *coli* MLST database). The clonal complexes (CC) were annotated using known clonal complex groups from the MLST database. This analysis involved a comparison of the allelic sequences of seven housekeeping genes (*adk*, *gyrB*, *icd*, *fumC*, *mdh*, *purA* and *recA*) as provided in Table S3. Serotypes of somatic (O) and flagellar (H) antigens were identified using Serotype Finder 2.0 [41] tool and the EcOH database using ABRicate [41,42].

### Variant calling analysis

The VCF file containing data from all 18 strains was analyzed using the SNPEff tool to identify genomic variants based on read data. First, the reads were aligned using the BWA-MEM tool to the reference genome (accession NC_000913.3) *E. coli* K-12 strain MG1655 using the following threshold settings: base quality 30, mapping quality 30, allelic frequency 0.75 and coverage 10. Assemblies were analysed using the contig-based workflow. Genomes were aligned with Nucmer against the reference strain, and SNPs were called using a delta filter and show-snp was distributed using the MUMmer package. SNP curation due to false-positive calls and SNPs located within repetitive or mobile regions in the reference (repeats, bacteriophages, plasmids and Insertion Sequence elements) were excluded.

SnpEff [43] generates a new VCF file containing the original data, and additional annotations of the possible variant effects are provided in Table S4. SnpEff also includes information on the type of mutation (transition or transversion), potential impact of the mutation (high, moderate, low or modifier), codon changes and aa changes.

### Screening of annotated genes against ABR databases

To understand the basis of antibiotic resistance gene functionality, we analysed sequence alignments using the CARD [44] database, MEGARes [45] database and ResFinder [46] database. The directory of the reference genome was created using the makeblastdb-in reference gene. The FastA -dbtype nucl-out database was aligned for each sample of *E. coli* using nt blast. Only drug-resistant genes with mutations were selected in Table S5a and S5b to generate plots for 18 *E. coli* strains. Mulitoperon plots were generated using the DNA Features Viewer [47], and detailed algorithms are provided in Python Script 1 ‘E.coliResistantViewer.py’. All images of the 18 genomes were combined into 1 central figure using a paint tool [48].

### Supervised machine learning

Machine learning methods were utilized to identify features in the 18 isolate genome sequences of the 18 isolates that were strongly correlated with resistance to each of the 17 selected antimicrobials (phenotypes). The process involves several key steps.

#### Feature table creation

Eighteen genomes were divided into overlapping 13 k-mers using a GenomeTester to create a feature table for all samples. The AMR phenotype of each sample (resistant, susceptible or intermediate) served as a class label, and intermediate phenotypes were excluded from the analysis. Qwing to class imbalance, a synthetic minority oversampling technique (SMOTE) was applied during classifier training to balance the class proportions in the dataset. The Python library was used for generating the overlapping 13 k-mers using the GenomeTester tool to compare the phenotype and genotype results of 18 *Klebsiella pneumoniae* from patients with sepsis.

Each isolate’s AMR phenotype, either resistant or susceptible, served as a class label, excluding intermediate phenotypes from the analysis. Due to class imbalance, SMOTE is a technique designed to address class imbalance, which is often a major issue in machine learning studies, particularly in medical datasets, and was applied during training to balance the datasets. The machine learning pipeline consisted of five main steps. First, feature selection involved discarding k-mers with a *P*-value >0.05 and retaining those with a *P*-value <0.05. These were input into an Extra Tree Classifier, and k-mers with a Gini feature importance above the overall mean were selected as shown in Table S6.

#### Classification and feature selection

Feature selection and classification were conducted using the Scikit-learn (Sklearn.model) package, employing a two-step approach to refine k-mer features. First, a chi-square test was used to discard k-mers with *P*-value >0.05, as these did not show a significant association with the AMR phenotype. Second, the remaining k-mers (*P*-value <0.05) were inputted into Extra Tree Classifier, a method based on randomized decision trees, to calculate the Gini feature importance. Only k-mers with a Gini importance above the overall mean were selected for further analyses. The selected k-mers served as input features for the classifiers, and their performance was evaluated using Python Script 2 ‘ChiSquare_FeatureSelection.py’. Metrics such as accuracy, area under the curve (AUC), sensitivity, precision, specificity and Cohen’s kappa value were calculated from 30 training runs for each antimicrobial, with Violin plots generated using the ggplot2 library.

#### Supervised machine learning testing

To evaluate the predictive power of the selected k-mers, multiple supervised machine learning methods were employed, including logistic regression (LR), linear support vector machine (L-SVM), radial basis function support vector machine (RBF-SVM), Extra Tree Classifier, random forest, AdaBoost, XGBoost, naïve Bayes, linear discriminant analysis and quadratic discriminant analysis. These models were implemented and evaluated using the Scikit-learn package, with the SMOTE technique applied during training in order to address class imbalance, as shown in Table S7. LR was prioritized because of its interpretability and simplicity, which are critical for deriving actionable insights into the relationship between genomic features and AMR phenotypes. Unlike more complex models such as random forest or SVM, LR allows for a straightforward interpretation of feature importance, aligned with the study’s goal of producing clinically relevant findings. Although complex models were tested for performance validation, they were not the primary focus because this study emphasized interpretability over predictive optimization.

#### Gene identification and selection

Gene identification was performed using a blastn search of whole-genome sequences to identify genes associated with each selected k-mers, as shown in Table S8. A blastn approach was applied to the 18 whole-genome sequence samples to identify the genes associated with each k-mer selected in the first step. Genes with a relative presence greater than 30% between the resistant and susceptible samples were selected for input into the cluster maps and gene-sharing networks. A heatmap was generated using the Python library to extract antimicrobial drug-resistant genes along with patient survival and non-survival outcomes or 17 classes of antibiotics from 18 *E. coli* infections. The performance metrics for the machine learning models included accuracy (TP+TN/{*P*+N}), sensitivity (true-positive rate {TP/P}), specificity (true-negative rate {TN/N}), area under the ROC curve (AUC) and Cohen’s kappa. Violin plots from the Seaborn package are used to illustrate the final prediction metrics.

To ensure sample representativeness and prevent overfitting, a wrapper backward selection approach was implemented, as shown in **Python Script “Wrapper_BackwardSelection.py”**. In each iteration, the least representative sample was removed, and the model was evaluated across all possible sample combinations. This process continued until the minimum sample size required for the SMOTE technique was achieved, ensuring both strong model performance and the retention of sample diversity

### Classification of phylogenetic analysis of *E. coli* using average nt identity cluster map

#### Genomic sequencing collection from the NCBI

The NCBI repository contained a diverse collection of *E. coli* reference genomes, including the K-12 strain MG1655 and associated metadata, which was used to establish a robust dataset for phylogenetic analysis.

#### Processing metadata and query genomes

The metadata files (10,647 entries) were processed by unzipping and renaming the contig files to .fna format. FastA files for the 18 query *E. coli* genomes were downloaded and manually renamed with the appropriate identifiers for consistency.

#### Calculation of average nt identity

Pairwise average nt identity (ANI) values were computed using tools such as FastANI calculator to compare the assembled genomes and evaluate the nt-level similarities. The resulting ANI matrix represents the genetic similarity between all genome pairs in the dataset.

#### Clustering and phylogroup classification

Hierarchical clustering was performed on the ANI matrix using a suitable clustering algorithm (e.g. agglomerative clustering). The clustering results were visualized using dendrograms and heatmaps in order to identify distinct genomic clusters corresponding to different phylogroups.

#### Creation of ANI cluster maps

ANI cluster maps were generated to visualize the phylogenetic relationships among genomes. Query *E. coli* phylogroups were annotated within the cluster maps to validate the dendrogram results. This approach accurately classified *E. coli* isolates into their respective phylogroups, providing valuable insights into their genetic diversity and potential pathogenicity, as provided in the Python Script 6 ‘Phylogroups/Metadata’.

### Computational tools and reference databases used for bacterial genome analysis

A comprehensive set of bioinformatic tools and curated databases was employed to analyse whole-genome sequences for AMR profiling, functional annotation, serotyping and structural genome characterization. Table 2 represents details of each computational resource used, the specific analytical purpose it served in this study and the corresponding citations. All tools were run using standard or recommended parameters unless otherwise specified.

**Table 2.** Computational bioinformatic tools used for genome annotation and functional analysis

Tool/database	Application in the study	Reference
ABRicate	Screened contigs for AMR and virulence genes using default parameters	[42]
CARD	Used with ABRicate to identify acquired AMR genes based on curated molecular models	[42,44]
Circos	Visualized genome features including AMR genes, GC content and SNP density	[40]
COG v12	Assigned functional categories to genes based on conserved orthologous groups	[35]
DNA Features Viewer	Visualized annotated features in genomes and plasmids	[47]
EcOH	*In silico* serotyping of *E. coli* O and H antigen loci	[41]
PANTHER v10 (GO)	Annotated genes to biological pathways and processes using GO terms	[36]
GenomeTester4	Determined k-mer presence/absence for comparative genomics	[58]
KEGG v4 (KAAS)	Reconstructed metabolic pathways using the KEGG Automatic Annotation Server	[34]
MEGARes v3.0	Identified AMR genes alongside CARD via ABRicate	[45]
Repeat Masker	Masked low-complexity and repetitive elements in assemblies	[32]
ResFinder	Confirmed acquired AMR genes, standalone or via ABRicate	[46]
Rfam v8	Annotated small RNAs using homology-based searches	[39]
RNAmmer v1.2	Predicted rRNA genes during genome annotation	[38]
Serotype Finder 2.0	Determined *E. coli* O:H serotypes from WGS data	[41]
Tandem Repeats Finder	Identified tandem repeat elements	[33]
tRNAscan-SE v1.3.1	Predicted tRNA genes and structures	[37]
SnpEff	Annotated SNPs and predicted their functional impact	[59]

## Results

### Epidemiological data

The demographic characteristics of the 18 patients with death and discharge outcomes in the context of sepsis and sepsis-associated comorbidity infections are listed in Table 3. Among the 18 cases, 6 were females, and 12 were male patients. The diverse disease conditions observed in these cases underscore the complexity of sepsis-related complications [2]. Kidney and urinary tract infection (UTI): acute kidney infection (AKI) was evident in five (27.78%), two cases manifested with rectal bleeding (11.12%), three cases exhibited AKI requiring haemodialysis (23.08%), renal failure was observed in two cases (11.12%), two cases had AKI along with urosepsis (11.12%), UTI cooccurring with urosepsis was identified in two cases (11.12%), one case presented with urosepsis (5.56%) and another case featured left hydronephrosis (5.56%) [3]. Meningitis and neurological complications: meningitis coupled with Parkinsonism was observed in five (27.78%) cases, two (11.12%) cases were associated with vasculitis and brain stem infarct, the other neurological complications, including one (5.56%) case each of obstructive sleep apnoea and (8.33%) disassociated disorder, and two (16.67%) cases displayed septic encephalopathy [4]. Pneumonia and respiratory distress: nine cases presented with pneumonia, where bronchopneumonia was diagnosed in three cases (50.0%), one case (5.56%) exhibited bronchopneumonia with type 1 respiratory failure, ventilator-associated pneumonia was identified in one case (5.56%), one case (5.56%) featured bronchial asthma, two cases were associated with bronchopneumonia and acute respiratory distress syndrome and subcutaneous emphysema were seen in one case (5.56%) [5]. Liver and pancreatic involvement: seven patients presented with liver and pancreatic disorders wherein liver acidosis was evident in three cases (16.67%), chronic liver disease was diagnosed in two cases (11.12%) and one case (5.56%) exhibited ascending cholangitis. Other comorbidities identified were diabetes mellitus in 13 patients (72.23%), hypertension in 8 cases (44.45%), sepsis and septic shock were documented in 5 (27.78%), 3 cases exhibited multiple organ dysfunction syndrome (MODS) (16.67%) and hypothyroidism was identified in 2 cases (11.12%) (see Table 3). Other conditions include right emphysematous pyelonephritis with diabetes mellitus, eustachian tube dysfunction, heart failure with a preserved ejection fraction and allergies.

**Table 3.** Demographic data and distribution of organ failure, organ dysfunction and outcome of the sepsis patients included in the study

Isolate descriptor	Source of infection	ICU	No. of days in ICU	Sex	Diagnosis	Antibiotic prophylaxis	The outcome of the cases
**CP091155_NEC1**	Community onset	CICU	4	M	Ischaemic heart disease; bronchopneumonia with septic shock	Combination of piperacillin and tazobactam	Death
**CP091156_NEC2**	Community onset	MICU	31	M	Organophosphorus poisoning with suicidal intention; right upper limb cellulitis; intermediate syndrome; bronchopneumonia with type 1 respiratory failure; AKI; and sepsis	Combination of piperacillin sodium and tazobactam sodium; meropenem; clindamycin	Discharge
**CP091157_NEC3**	Hospital onset	MICU	3	M	UTI with urosepsis; AKI on HD; type 2 DM; metabolic acidosis with dyselectrolytaemia; and Parkinson’s disease	Combination of piperacillin and tazobactam; clindamycin	Discharge
**CP091158_NEC4**	Community onset	MICU	9	F	Healing ulcer over right lower limb and bronchopneumonia	Clindamycin, sulbactam sodium and cefoperazone sodium	Discharge
**CP091159_NEC5**	Hospital onset	RICU	7	F	Right lower lobe pneumonia; AKI; type 2 DM and HTN; subcutaneous emphysema; and dissociative disorder	Meropenem; combination of piperacillin and tazobactam	Death
**CP091160_NEC6**	Hospital onset	MICU	1	M	Septic encephalopathy with septic shock and MODS; AKI; and type 2 DM	Ceftriaxone; doxycycline; tetracycline; meropenem; and levofloxacin	Death
**CP091161_NEC8**	Hospital onset	RICU	10	M	Bronchopneumonia; type 2 DM and HTN; and severe sepsis	Piperacillin sodium and tazobactam sodium	Death
**CP091298_NEC9**	Community onset	RICU	5	F	Ventilator-associated pneumonia; allergies; AKI; urosepsis; septic shock; MODS; and Parkinson’s disease	Ceftriaxone and meropenem	Death
**CP091162_NEC10**	Community onset	MICU	9	F	Type 2 DM; sepsis with septic shock; lower CBD structure cholangitis; renal failure; and left hydronephrosis; lower CBD structure cholangitis; renal failure; and left hydronephrosis	Piperacillin sodium and tazobactam sodium	Death
**CP091163_NEC11**	Community onset	MICU	23	M	Type 2 DM; HTN; DCM-35%; chronic SDH S/P BURRHOLE; Parkinsonism; bedsores with sepsis; and septic shock	Metronidazole; piperacillin sodium and tazobactam sodium; cefuroxime	Discharge
**CP091164_NEC12**	Hospital onset	MICU	15	M	Urosepsis; septic encephalopathy; AKI, anuric, severe metabolic acidosis; Parkinson’s disease; and thrombocytopenia	Meropenem sulbactam; polymyxin E	Discharge
**CP091165_NEC13**	Hospital onset	MICU	15	M	Rectal bleeding; UTI with urosepsis; AKI; and septic shock	Piperacillin sodium and tazobactam sodium, meropenem	Discharge
**CP091166_NEC14**	Community onset	MICU	15	F	Left pyelonephritis; type 2 DM and eustachian tube dysfunction	Piperacillin sodium and tazobactam sodium; meropenem	Discharge
**CP091167_NEC16**	Hospital onset	MICU	11	M	Right emphysematous pyelonephritis with DM, hypothyroidism and fever	Azithromycin	Death
**CP091168_NEC17**	Community onset	SICU	4	M	Cholelithiasis with CBD and sepsis with AKI	Piperacillin sodium and tazobactam; meropenem	Discharge
**CP091169_NEC18**	Community onset	MICU	8	M	UTI with urosepsis; hypoglycaemia secondary to OHA; type 2 DM and HTN; Churg–Strauss syndrome; vasculitis-brain stem infarct; bronchial asthma; and right renal calculi	Cefuroxime; meropenem	Discharge
**CP091170_NEC19**	Community onset	RICU	1	M	Sepsis with septic shock; bronchopneumonia with ARDS with type 1 respiratory failure; AKI; and chronic liver disease	Meropenem	Death
**CP091171_NEC20**	Community onset	RICU	41	F	Bronchopneumonia; repeat RT-PCR - negative; type 2 DM and HTN; AKI with sepsis; and septic shock	Cefuroxime; meropenem	Discharge

BURRHOLE, a burr hole is a small cavity drilled into the skull; CBD, corticobasal degeneration; DCM, dilated cardiomyopathy; DM, diabetes mellitus; HD, haemodialysis; HTN, hypertension; RT-PCR, Reverse-Transcriptase Polymerase Chain Reaction.

### Antibiotic phenotype profiling

The ESBL phenotype was identified using the double-disc synergy method on Mueller–Hinton agar plates by placing ceftriaxone discs 20 mm apart from the amoxicillin and clavulanic acid discs. The apparent inhibition zone around the ceftazidime or ceftriaxone discs near the clavulanic acid-containing disc confirmed the ESBL production (see Table 1).

### Mapping the positions of AMR and virulence genes in 18 *E. coli* genomes using MUMmer 2 Circos

**Fig. 1.**
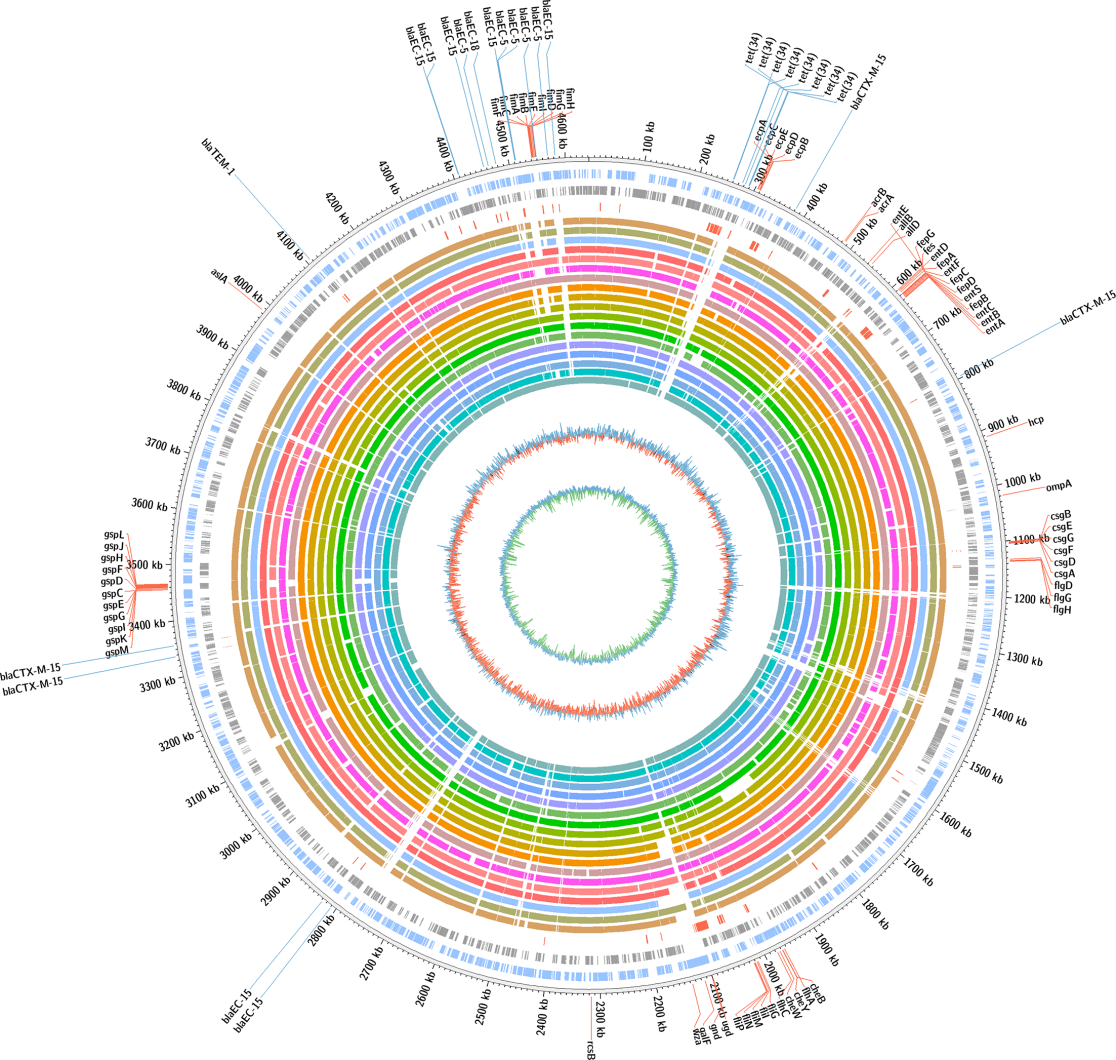
*E. coli* strain K-12 (NC_000913.3) microbial nt essential local alignment search tool (blast) analysis and annotation. The % similarity of all 18 genomes was analysed. Afterwards, the metadata was plotted in a circular map using Circos software. The first outer and inner ring represents the reference genome, with positive and negative nt sequences of 46,000 kb. The second ring illustrates the negative ORF gene strands. The third ring indicates the positive strands of the genomic metadata. The fourth ring highlights unique genes that exhibit multidrug resistance and virulence sequences. Additionally, the percentage identity of the reference genome was also included. The fifth to twenty-second rings correspond to the sepsis and sepsis-associated case-specific 18 *E. coli* genomes. The twenty-third ring represents the GC contents. The twenty-fourth ring illustrates GC-skew distribution=(G-C)/(G+C). Blue and green indicate negative and positive graph values, respectively. The respective colour code and STs along with serotypes of 18 genomes are provided in a small box.

The Maximum Unique Match finder (MUMmer) is a software package designed for the rapid alignment of large DNA or protein sequences. The MUMmer high-speed alignment was optimized for the entire genome globally, locally and for unique matches. Circos is a visualization tool designed to represent complex datasets in an interactive circular layout. When used together, MUMmer generates alignment data that Circos can transform into a detailed and visually appealing representation. Alignment of multiple genomes to detect conserved regions, with functional genes of various categories given in Table 4, and detailed alignments are shown in Fig. 1.

**Table 4.** Summary of antibiotic resistance genes, VFs and STs identified in 18 *E. coli* genomes

Property	Description
**Antibiotic resistance genes**	
*blaEC*	100% (18 genomes)
*blaEC-15*	38.89% (7 genomes)
*blaEC-5*	27.78% (5 genomes)
*blaEC-18*	5.56% (1 genome)
*blaCTX-M-15*	22.23% (4 genomes)
*blaTEM-1B*	5.56% (1 genome)
*tet-34*	44.45% (8 genomes)
**VFs**	100% (18 genomes)
**Fimbrial genes**	*ecpA*, *ecpB*, *ecpC*, *ecpD*, *ecpE*, *fimH*, *fimG*, *fimF*, *fimD*, *fimI*, *fimE*, *fimB*, *fimA*, *fimC*
**Efflux pump genes**	*acrB*, *acrA*
**Iron uptake and metabolism genes**	*entE*, *allB*, *allD*, *fepG*, *fes*, *entD*, *fepA*, *entF*, *fepC*, *fepD*, *entS*, *fepB*, *entC*, *entB*, *entA*
**Curli and flagellar genes**	*csgA*, *csgB*, *csgE*, *csgF*, *csgG*, *flhC*, *flgD*, *flgG*, *flgH*, *cheB*, *flhA*, *fliP*, *cheY*, *cheW*, *fliG*, *fliM*, *fliN*, *fliI*
**Type II secretion system genes**	*gspL*, *gspF*, *gspD*, *gspC*, *gspG*, *gspE*, *gspJ*, *gspI*, *gspM*, *gspH*, *gspK*
**Additional genes**	*ompA*, *ugd*, *gnd*, *galF*, *wza*, *rcsB*
**STs and serotypes**	
ST69	Serotypes O15:H18 and O17:H18
ST2851	H18 serotype; O-group serotype unidentified
ST405	Serotype O102:H6
ST131	Serotype O25:H4
ST361	Serotype O9:H30
ST167	Serotype H9; O-group serotype unidentified
ST1287	Serotype O9:H9
ST12709	Serotype unknown
**ESBL-producing isolates**	Identified in ST1284, ST2851, ST167, ST361, ST405, ST8346, ST450 and ST131

### Generation of heatmap of virulence genes of 18 *E. coli* using the virulence finder database

**Fig. 2.**
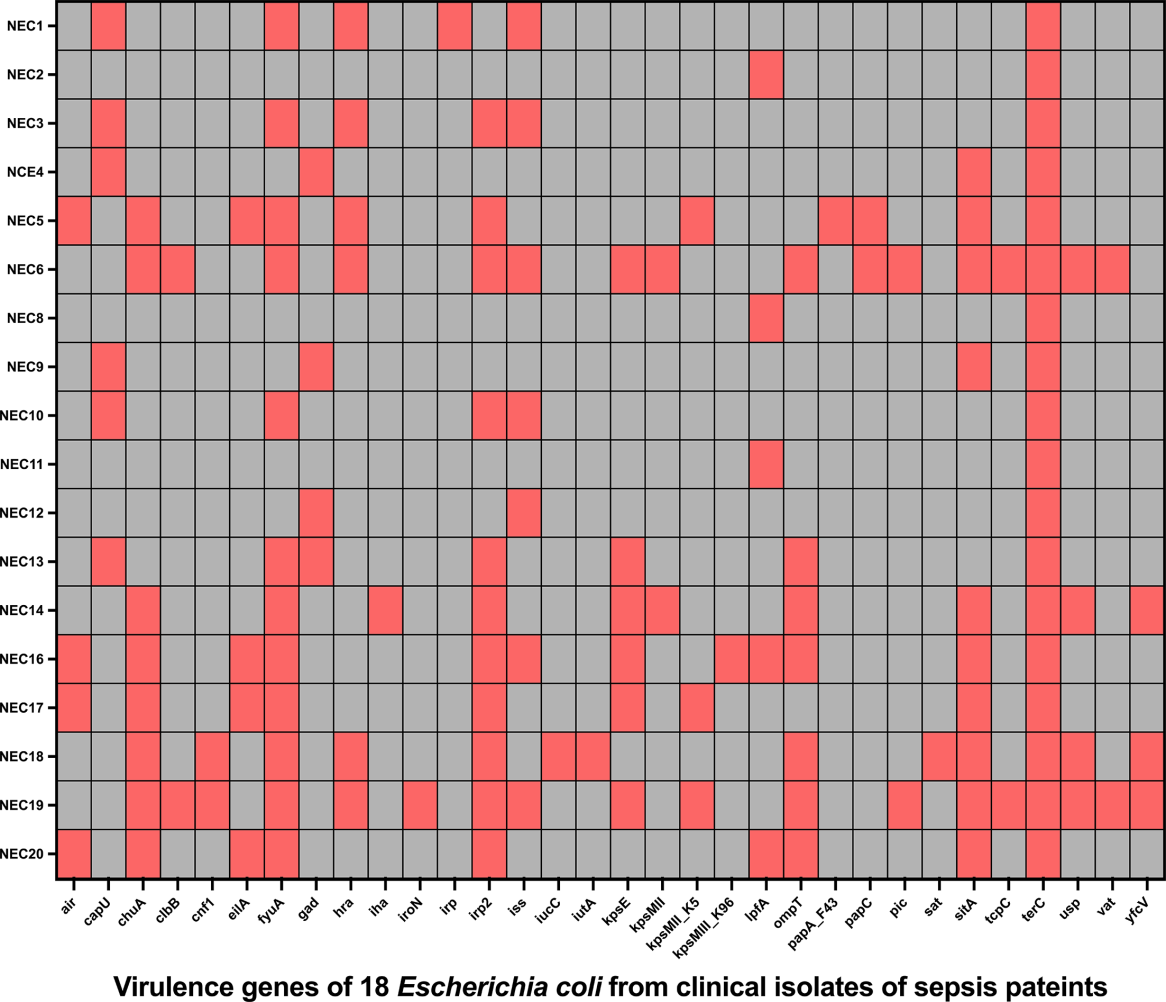
Heatmap of highly expressed VFs of 18 *E. coli* genomes from sepsis patient.

This study was conducted to determine the number of genes in adults with sepsis and revealed contradictory findings regarding the impact of VFs on the suitability of empiric antimicrobial therapy and sepsis outcomes. A few of the virulence potential genes were highly expressed as shown in Table 5. A heatmap of the VFs of 18 *E. coli* genomes from patients with sepsis is shown in Fig. 2.

**Table 5.** Distribution of virulence genes in 18 *E. coli* genomes

Gene	Description	Prevalence (%)
*air*	Aerotaxis receptor (aer expression regulated by FlhD/FlhC)	22.2 (4 genomes)
*capU*	Putative hexosyltransferase CapU	27.7 (5 genomes)
*chuA*	TonB-dependent haem/haemoglobin receptor ChuA/ShuA	44.4 (8 genomes)
*clbB*	Colibactin hybrid non-ribosomal peptide synthetase	11.1 (2 genomes)
*cnf1*	New gene	11.1 (2 genomes)
*eilA*	HilA/EilA family virulence transcriptional regulator	22.2 (4 genomes)
*fyuA*	Siderophore yersiniabactin receptor FyuA	66.6 (12 genomes)
*gad (gapA*)	Glyceraldehyde-3-phosphate dehydrogenase A	22.2 (4 genomes)
*hra*	Glyoxylate/hydroxypyruvate reductase A (Thra)	33.3 (6 genomes)
*iha*	Iha adhesin	5.5 (1 genome)
*iroN*	TonB-dependent siderophore receptor protein	61.1 (11 genomes)
*irp*	Yersiniabactin non-ribosomal peptide synthetase	61.1 (11 genomes)
*iss*	Prophage lipoprotein BorD	38.8 (7 genomes)
*iucC*	NIS family aerobactin synthetase IucC	5.5 (1 genome)
*iutA*	New gene	5.5 (1 genome)
*kpsE*	Capsule polysaccharide export inner-membrane protein KpsE	33.3 (6 genomes)
*kpsMII*	Microcin C and K1 capsule group 2	11.1 (2 genomes)
*ipfA*	Adhesins to biofilm	27.7 (5 genomes)
*ompT*	Omptin family outer membrane protease OmpT	38.8 (7 genomes)
*papA_F43*	ATP synthase F1 complex subunit *α*	5.5 (1 genome)
*papC*	ATP synthase F1 complex subunit *γ*	11.1 (2 genomes)
*pic*	DNA damage-binding protein Pic	11.1 (2 genomes)
*sat*	Acetate/succinate: H+symporter involved in acetate homeostasis	5.5 (1 genome)
*sitA*	Iron/manganese ABC transporter substrate-binding protein SitA	55.5 (10 genomes)
*tcpC*	Pseudogene	11.1 (2 genomes)
*terC*	Resistance to tellurite	100 (18 genomes)
*usp*	Universal stress protein activator	22.2 (4 genomes)
*vat*	Vat family streptogramin A O-acetyltransferase gene	11.1 (2 genomes)
*yfcV*	Putative fimbrial protein YfcV	16.6 (3 genomes)

### Integrated SNP analysis in Antibiotic resistance genes leveraging CARD, MEGARes and ResFinder databases

**Fig. 3.**
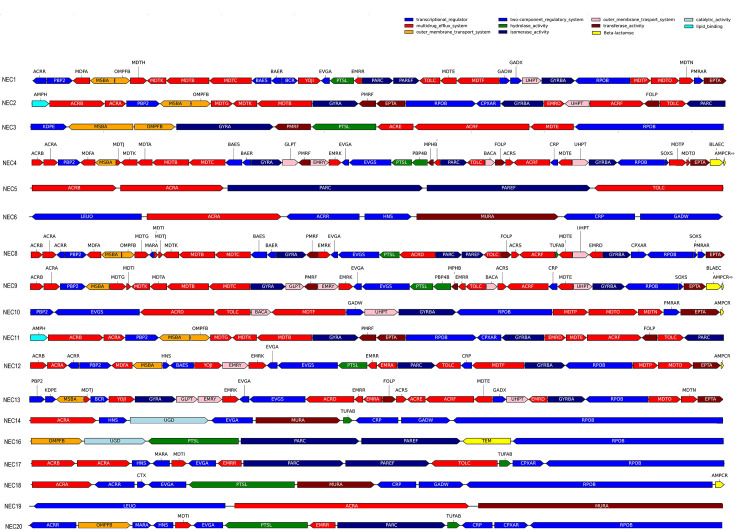
Mutational analysis of antibiotic-resistant genes in 18 *E. coli* genomes. Resistance genes were matched in the CARD and MEGARes databases identified from individual scaffolds. The 11 main functional properties (with high-impact mutational features) are plotted to determine unique variations.

We further continued our study to understand the relatedness of operons of isolates in our study. The results were compared within the number of core gene SNPs from variant calling files of 18 *E. coli* genomes combining drug-resistant sequences from three different databases: CARD [44], ResFinder [46] and MEGARes [45]. Alignment was performed for 18 *E. coli* genomes using nt blast: blastn -query. A total of 550 antibiotic-resistant genes were identified across the 18 *E. coli* genomes. These genes were classified based on their resistance to various antibiotic classes, such as beta-lactams, quinolones, aminoglycosides, tetracyclines and sulphonamides. All determinants were matched to genes in the Pathogen Detection Resources of the NCBI with a similarity of at least 99% and then further named and classified according to the matched gene information in Fig. 3, which included transcriptional regulator genes, two groups of outer membrane transport system genes, two-component regulatory system genes, catalytic activity genes, multidrug efflux system genes, hydrolase activity genes, transferase activity genes, lipid-binding genes, aminoglycoside-modifying enzyme genes, isomerase activity genes and beta-lactamase genes with related enzymes. Only drug-resistant genes with mutations were selected to generate plots for the 18 *E. coli* strains.

### Structural analysis and functional annotation of *E. coli* pathogenicity island

**Fig. 4.**
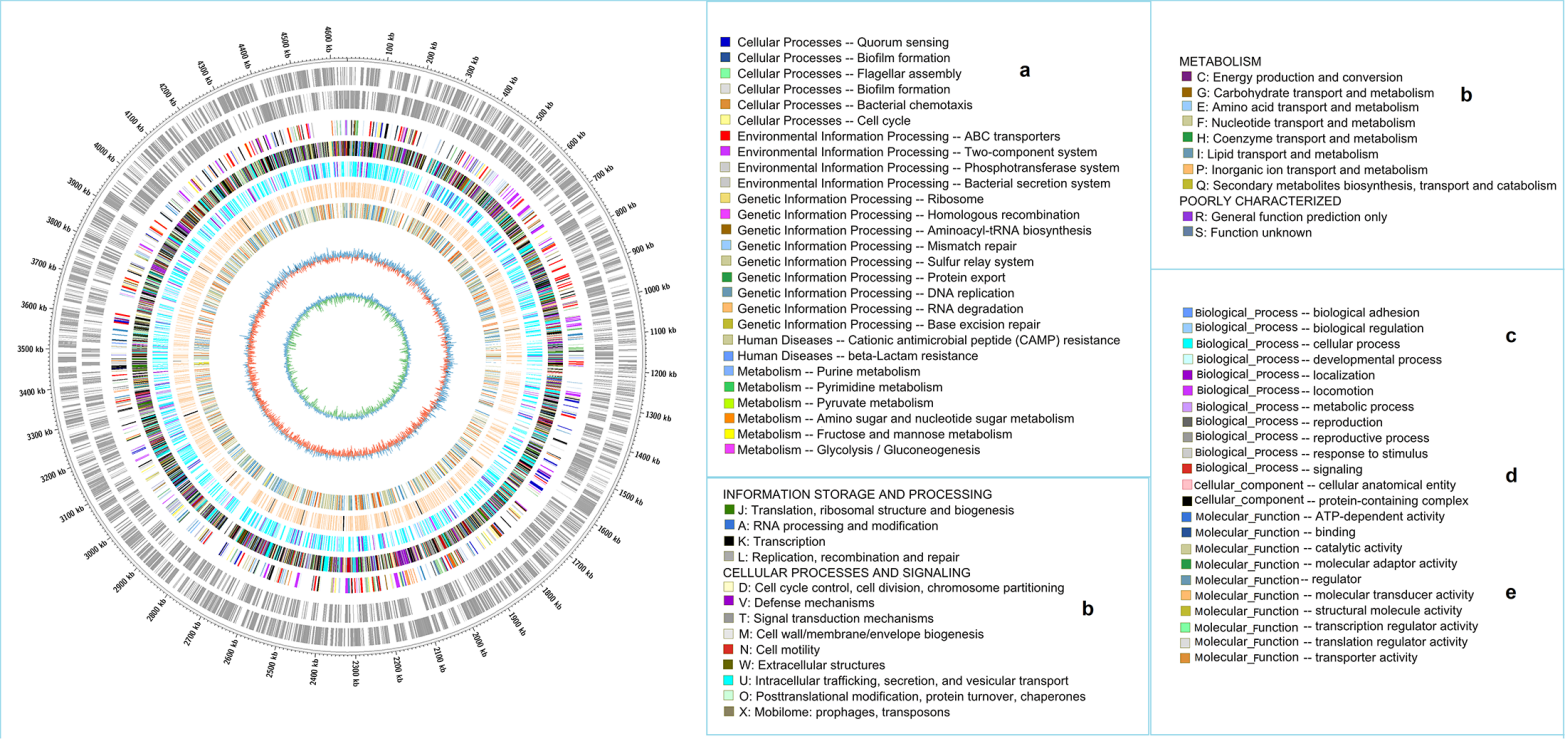
Circular plot representation of the metabolic processing of the *E. coli* genome. The outer and inner sides of the circles represent the positive and negative strands of the genome, respectively. The first circle corresponds to the reference genome, representing the *E. coli* strain K-12 (NC_000913.3). The second to fourth circles indicate the identified genes from KEGG, COG and GO functions. Each colour denotes the functional molecular classification of gene positions within the *E. coli* genome. The sixth circle illustrates the GC content. The seventh circle represents the GC-skew distribution, calculated as GC-skew=(G−C)/(G+C). Blue and green indicate negative and positive graph values, respectively.

The Circos visualization method illustrated the relationships and patterns among different biomolecule groups within the 18 *E. coli* genomes. Group (a) included genes associated with six cellular and four environmental functions (biofilm formation), nine genotype processes, two human diseases, two nt metabolism, one aa metabolism and two nt sugar metabolisms, one fructose and one mannose metabolism and glycolysis or gluconeogenesis. Group (b) comprised genes involved in replication, DNA recombination and repair mechanisms, transcription and transitional biogenesis, cell signalling, cell division, supercoiling, condensation, defence mechanisms, signal transduction and cell wall and cell membrane biogenesis, as well as extracellular structure, intracellular trafficking, vesicular transport secretion, cell motility, post-translational modifications, protein turnover, chaperone protein activation, mobility (prophages and transposons) and energy production and conservation. It also included genes for carbohydrates; aa; nt; coenzymes; lipids; inorganic ion transport and metabolism; and secondary metabolite biosynthesis, transport and catabolism. Group (c) comprised genes involved in biological processes, such as adhesion, cell regulation, cellular development, localization, locomotion, reproduction and stimulus responses. Group (d and e) included genes involved in cellular processes and molecular functional properties. The fifth circle displays the ncRNA results shown in Fig. 4.

### A computational framework for merging machine learning and genome metabolic models

To uncover the genomic features associated with specific antibiotic-resistant phenotypes, a computational workflow was developed that integrates machine learning with genome metabolic models. The workflow involved the following steps: (1) Categorization of genome content and AMR profiles: genome sequence and AMR profiles were classified to establish a foundation for analysis. (2) Sequence redundancy reduction: the CD-hit tool was used to cluster sequences, reduce redundancy and improve the computational efficiency of the subsequent analyses. The results are presented in Table S9. (3) Retrieval and concatenation of coding sequence (CDS): CDS from FastA files were extracted and concatenated for all *E. coli* isolates. (4) K-mer similarity confirmation: generated k-mers were filtered to confirm ≥90% sequence similarity. (5) blast-based sorting of k-mers: significant k-mers were sorted based on similarity and percentage identity from the blast results. The k-mers were mapped to AMR sequences from 18 *E. coli* genomes using blastn and aligned against databases, such as CARD, ResFinder and NCBI AMR Finder (Table S10).

We used 10 supervised learning classifiers to predict the resistant and susceptible strains for each of 16 antimicrobials (see Table 1). These include LR, L-SVM, RBF-SVM, Extra Tree Classifier, random forest, AdaBoost, XGBoost, naive Bayes, linear discriminant analysis and quadratic discriminant analysis. Table S11 provides a detailed summary of the classifiers’ performance. A two-step feature selection method was applied to reduce the number of features analysed by the classifiers: (a) k-mers with a *P*-value >0.05 from a chi-square test were discarded, and (b) the remaining k-mers were input into an ~Extra Tree Classifier to select features with a high Gini importance. This streamlined the analysis and ensured that only statistically significant features contributed to classification. Owing to the small sample size, eight antimicrobials (ampicillin, cefepime, ceftriaxone, cefuroxime, ciprofloxacin, nalidixic acid, tigecycline and colistin) were excluded from subsequent analysis, as cross-validation and SMOTE modelling could not be applied. For the remaining eight antimicrobials, classification results highlighted the ability to effectively discriminate resistant and susceptible strains by leveraging known and novel genetic determinants using Python Script 4 ‘AMR_FeatureSelection_Classifier.py’.

**Fig. 5.**
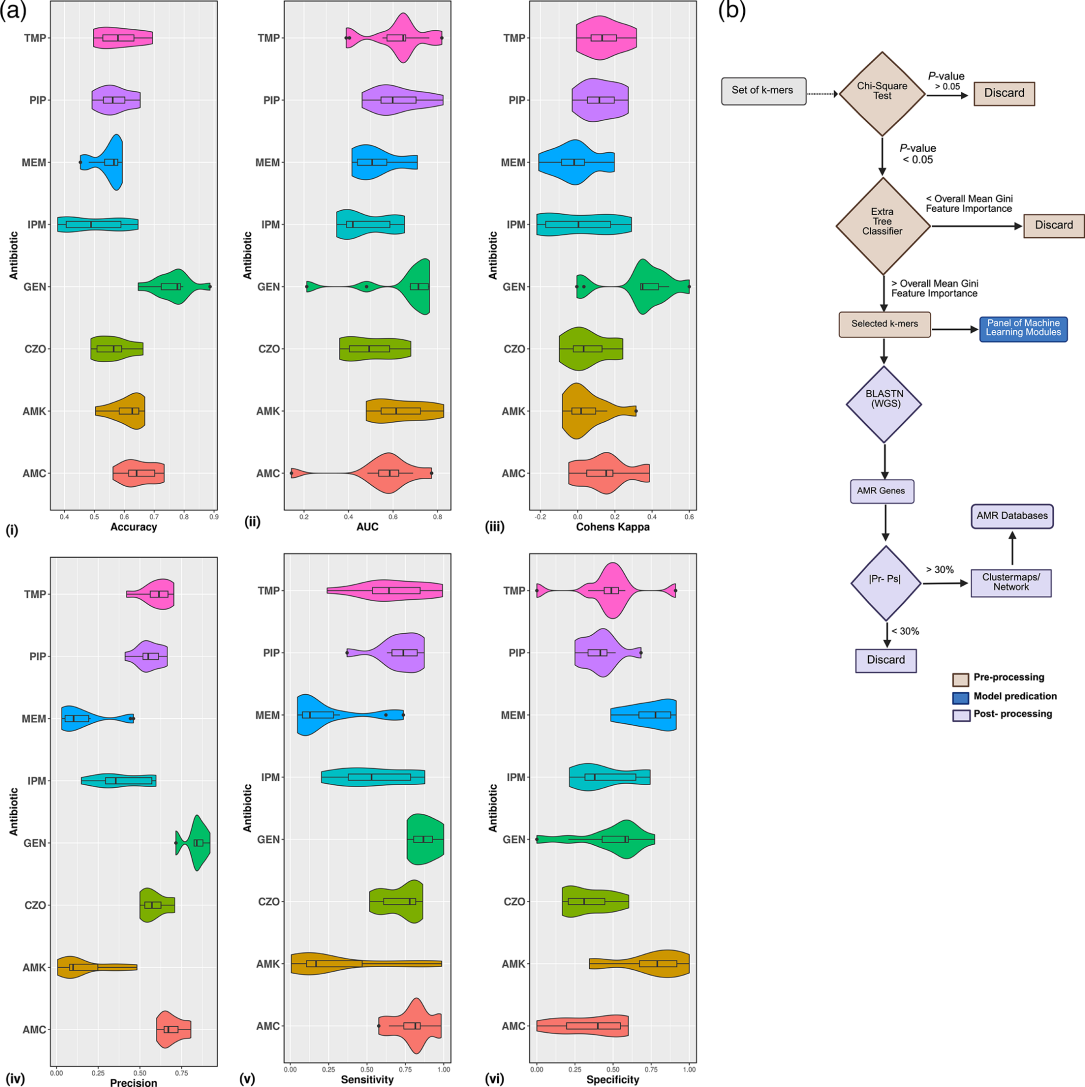
Supervised machine learning prediction of AMR signature profiles to eight antimicrobials in the *E. coli* genomes. Prediction performance results of the RBF-SVM classifier that achieved the best performance among the three investigated are shown. Five performance indicators have been used to evaluate the classification (a): (i) accuracy, (ii) AUC, (iii) sensitivity, (iv) precision, (v) specificity and (vi) Cohen’s kappa value from 30 training runs for each antimicrobial. The scores for each performance metric are indicated in the Y-axis. Predictive models were generated to classify the resistance vs. susceptibility profiles of eight different antimicrobials (X-axis): amoxicillin/clavulanic acid (AMC), amikacin (AMK), cefoperazone/sulbactam (CZO), gentamicin (GEN), imipenem (IPM), meropenem (MEM), piperacillin/tazobactam (PIP) and trimethoprim/sulfamethoxazole (TMP). (b) Flow diagram showing the machine learning pipeline, including feature selection (brown), classification (dark blue) and post-processing (light blue).

Trimethoprim/sulfamethoxazole, piperacillin/tazobactam and amikacin achieved AUC scores >0.8, with trimethoprim displaying the highest AUC score of 0.819±0.069. LR was performed comparably to the other classifiers, achieving robust sensitivity, specificity and precision metrics (Fig. 5 and Table S12). Trimethoprim achieved an accuracy of 0.694±0.071, sensitivity of 0.861±0.105 and a Cohen’s kappa score of 0.316±0.112. Precision score ranged from 0.703±0.084 to 0.991±0.03.

**Fig. 6.**
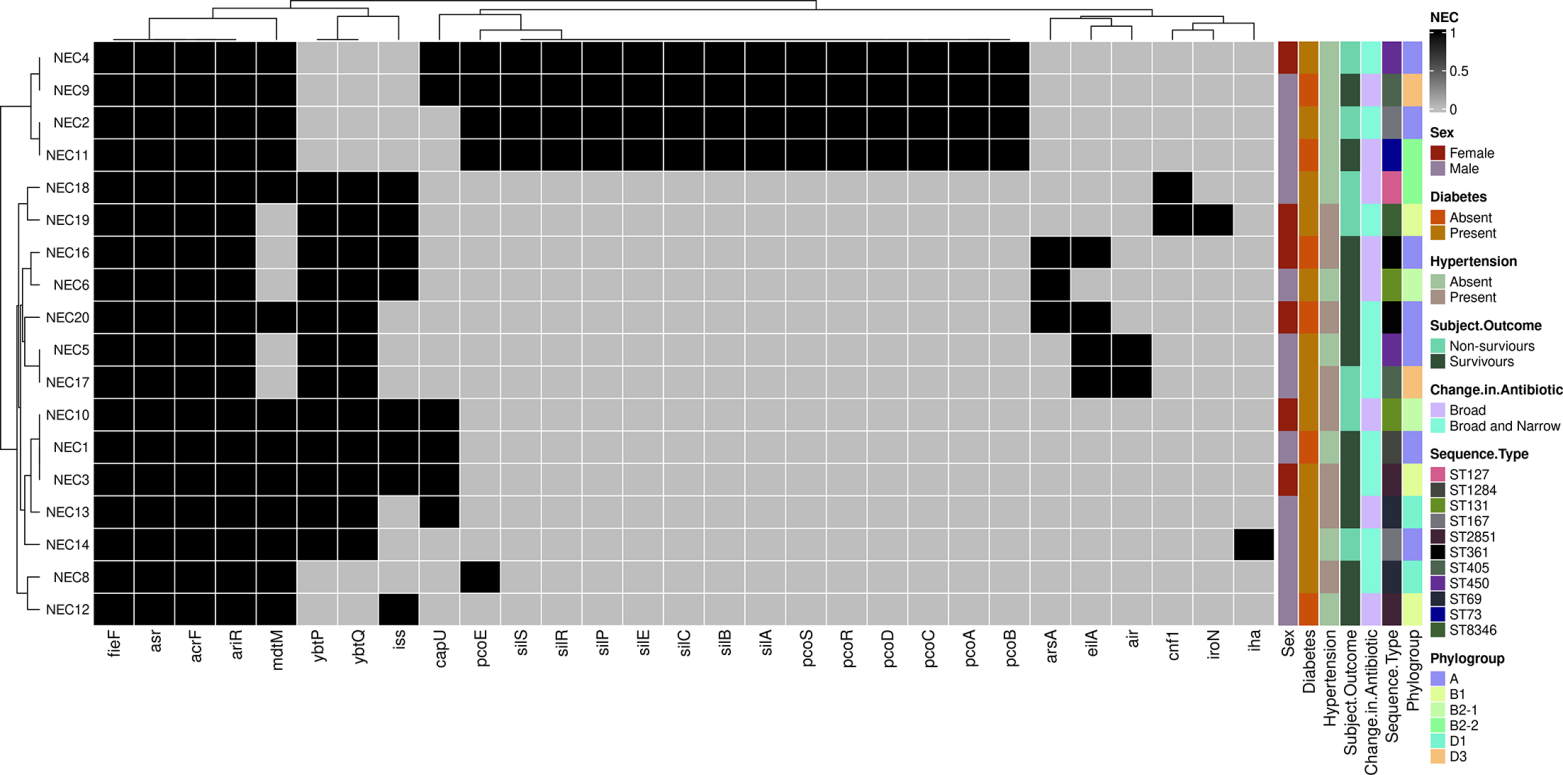
Display of ranked clustered commonly found genes of AMR associated with phylogenetic hierarchy of 18 genomes of *E. coli* using R programme. This figure displays a heatmap depicting the significant findings of our AMR analysis within individual 18 *E. coli* sepsis-causing genomes. Additionally, the figure includes detailed demographic information on sepsis cases, such as sex, comorbidities (including type 2 diabetes and hypertension), ICU admission outcomes, changes in antibiotic treatments during hospitalization, genomic STs and their corresponding phylogroups.

The test performance curves indicated that the classifier performance plateaued, suggesting a significant impact of increasing sample size on improving predictions across all antimicrobial classes. To identify genes critical for AMR, we prioritized the k-mers linked to genes with the largest discrepancies between resistant isolates (Pr) and susceptible isolates (Ps). Genes with |Pr-Ps| differences ≥30% were flagged as significant contributors. In total, 193 unique genes were identified across all models, including 29 AMR-related genes from the CARD and ResFinder databases, as shown in Fig. 6. Further analysis revealed that 678 genes were significantly associated with the resistance phenotypes. These genes were classified based on their roles in beta-lactam, cephalosporins, carbapenems, penicillins (e.g. amoxicillin and methicillin) and cephamycins. These classes of antibiotics, which are widely used in ICU settings, were selected based on their clinical relevance provided in Table S13. Heatmaps illustrating the distribution of resistance profiles for the 17 antimicrobial agents were generated using Python Script 5 ‘AMR_Heatmaps_Generator.py’ in R (Fig. S4). These visualizations highlight the functional and phenotypic variability of AMR across isolates, highlighting the genomic basis of resistance in sepsis-related *E. coli* infections.

### Dendrogram maps depicting phylogenetic tree analysis of *E. coli* genomes available from the NCBI repositories

**Fig. 7.**
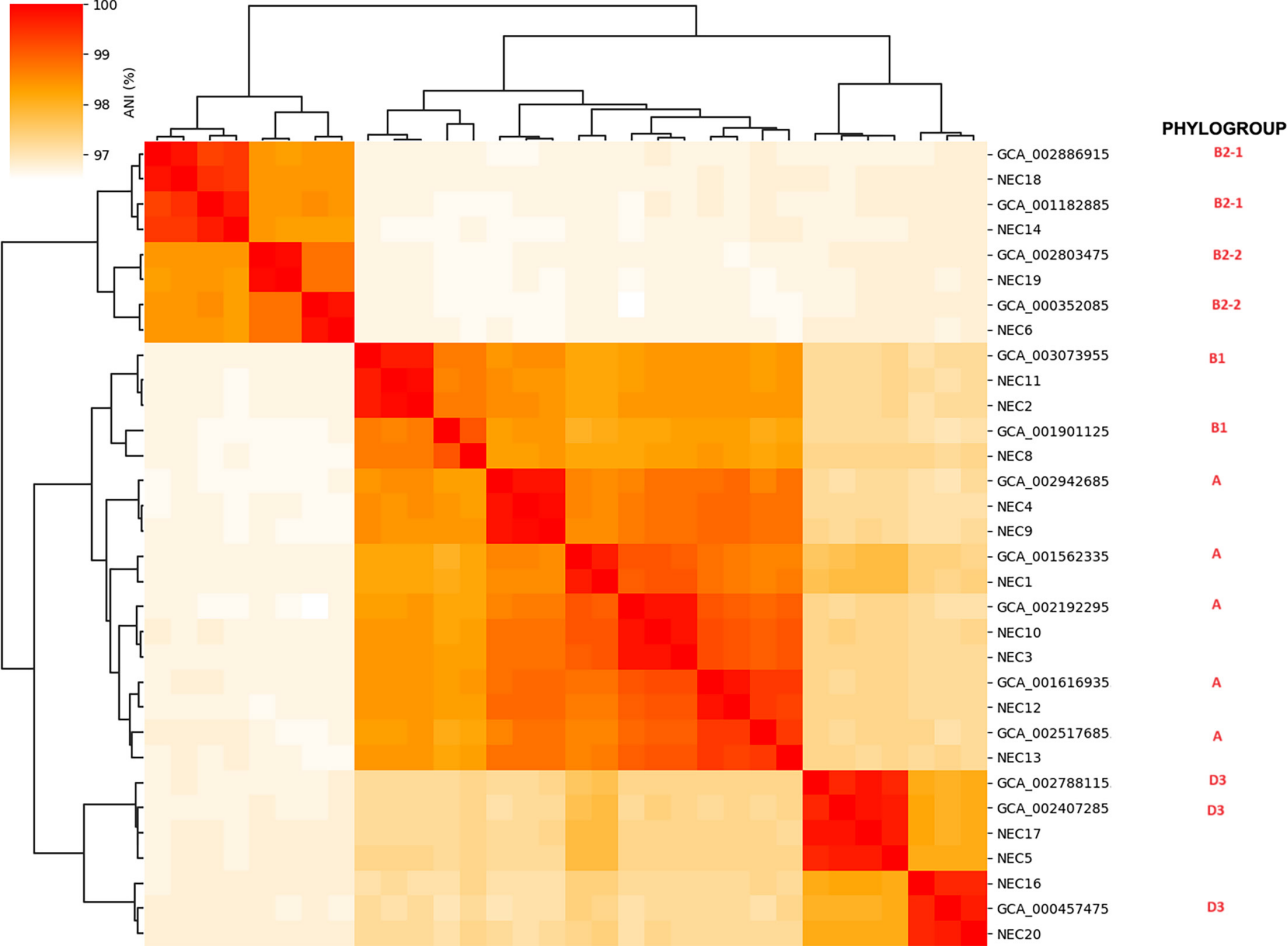
Dendrogram heatmap depicting phylogenetic tree analysis and classification of phylogroups of 18 whole genomes of *E. coli* using ANIclustermap hierarchical clustering based on ANI depicts the phylogenetic relationships and phylogroup classification among 18 *E. coli* genomes. The dendrogram illustrates genomic similarity, while the accompanying heatmap represents ANI percentage values, clustering group-level taxonomic resolution.

Classification of the phylogroups of 18 *E. coli* genomes using the ANI cluster map provided valuable insights into their genetic relationships and evolutionary divergence. ANI clustering, a robust method for assessing genomic similarity, uses a threshold of ≥95% ANI to group bacterial strains within the same species. By visualizing the phylogenetic relationships among the 18 *E. coli* genomes, ANI clustering enabled precise grouping into distinct phylogroups (A, B1, B2-1, B2-2 and D3; Fig. 7). This analysis highlights the correlation between genomic diversity and functional and phenotypic traits such as VFs, antibiotic resistance and ecological niches. For example, phylogroups such as B2 are often associated with extraintestinal pathogenic *E. coli* strains that cause serious infections. In contrast, phylogroups A and B1 are more commonly linked to commensal or environmental strains with a lower pathogenic potential. Identifying these phylogroups is critical to elucidate the evolutionary trajectories, pathogenic potential and transmission patterns of *E. coli*. Our findings have practical implications for future research. ANI-based phylogroup classification facilitates the identification of emerging strains of public health concern and aids in targeted interventions for infection control and antibiotic stewardship programmes. These insights will contribute to improved public health outcomes by enabling precise monitoring, prevention and treatment strategies for *E. coli*-related infections.

## Discussion

### WGS in AMR surveillance and management

WGS transforms AMR surveillance and management by providing unprecedented insights into the genetic mechanisms underlying resistance and pathogen evolution. Its integration into national and global surveillance frameworks, such as the WHO’s Global Antimicrobial Resistance Surveillance System (GLASS) and regional initiatives, has enabled proactive strategies to address the AMR crisis. WGS facilitates high-resolution comparisons of AMR genes, STs and plasmid content across borders, thereby promoting collaborative data sharing and cross-referencing. This approach supports the early detection of emerging resistance trends, identification of global and regional transmission patterns and creation of a comprehensive AMR genomic repository.

WGS-driven surveillance can inform public health policies by identifying resistance determinants and their prevalence in healthcare and community settings. These data will enable the development of evidence-based antibiotic stewardship programmes tailored to local AMR profiles. Policies on empirical antibiotic use can be refined to reflect resistance trends, optimize treatment efficacy and reduce the selective pressure that drives resistance. Furthermore, WGS provides critical insights into detecting MDR organism outbreaks. By pinpointing the clonal spread of AMR strains within healthcare facilities or across regions, WGS facilitates source tracing and targeted interventions, thereby enhancing response efficiency.

WGS’s standardized genomic analyses ensure consistent and reproducible pathogen characterization, allowing robust comparisons of resistance trends over time and between regions. This standardization bridges the gaps caused by variations in phenotypic testing methodologies, bolstering global AMR monitoring efforts.

### AMR and VF potential in *E. coli* clinical isolates

This study investigated the AMR and VF potential of 18 *E. coli* isolates from patients with sepsis admitted to a tertiary care hospital in India. The isolates were resistant to cefuroxime (89%), ciprofloxacin (84%), ampicillin (78%) and ceftriaxone (78%). Similar resistance trends have been reported in previous studies [49]. For example, in a Nigerian tertiary care hospital, 80.4% and 94.3% of *E. coli* strains were resistant to ceftriaxone and ampicillin, respectively [33]. Similarly, an Indonesian study reported resistance rates of 86% to cefuroxime and 52% to ciprofloxacin in *E. coli* strains [50].

The high resistance rates observed may result from the empirical use of antibiotics in patients with sepsis, which is often initiated without a confirmed diagnosis or adjusted for organ failure. The misuse of antibiotics exacerbates the development of AMR in *E. coli* and contributes to adverse ecological effects. Poor healthcare infrastructure in developing regions such as India, Pakistan, Indonesia and Nigeria also plays a critical role in the emergence of AMR pathogens, including *E. coli* [51].

### ST and serotype diversity in *E. coli* isolates

This research comprehensively analysed STs and serotypes among the isolated *E. coli* genomes, revealing a wide diversity of strains, including ESBL-producing variants. Among these, ST131 was identified, which is frequently associated with UTIs. This strain is associated with both acute and chronic conditions. Its periodic introduction from the community to hospital settings highlights the dynamic exchange of pathogenic *E. coli* strains [52]. The coexistence of diverse *E. coli* lineages ranging from commensal to highly pathogenic strains reflects an organism’s genetic adaptability to selective pressure. This diversity impacts clinical management and highlights the importance of continuous genomic surveillance to monitor the emergence of new STs and serotypes. The early detection of high-risk strains can inform tailored infection control policies and optimize patient outcomes. By integrating serotype and ST analyses into infection control strategies, healthcare systems can better address the dynamic challenges posed by *E. coli* in both community and hospital settings.

### Genomic insights into AMR determinants

Using bioinformatic tools, we predicted antibiotic-resistant genes and VFs in clinical *E. coli* isolates, offering valuable insights into AMR. Key findings included the elucidation of ESBL class C *β*-lactamase genes (e.g. *blaEC* subtypes: *blaEC-15*, *blaEC-5*, *blaEC-18*, *blaCTX-M-15* and *blaTEM-1B*) and multidrug efflux pump genes (e.g. *mdf(A)* and *acrF*, both 100%). ESBL production confers resistance to third- and fourth-generation cephalosporins, severely limiting treatment options and worsening the disease severity [53].

### Bioinformatic analysis of *E. coli* pathogenicity islands

Bioinformatic analysis of the *E. coli* pathogenicity island, visualized using Circos, revealed diverse biomolecular groups crucial for bacterial virulence and pathogenicity. These included genes associated with cellular and environmental functions (e.g. quorum sensing, biofilm production and bacterial chemotaxis), human diseases (e.g. *β*-lactam resistance) and biological processes (e.g. adhesion and signalling). Notably, virulence genes encoding two toxins (*vat* and *pic*), one protectin (*iss*) and various adhesins (*air*, *ipfA*, *fdeC* and *yagW/ecpD*) were identified. These genes enable *E. coli* to adhere to the host tissues, evade immune responses and adapt to environmental changes, thereby contributing to the development of severe sepsis [54]. While these virulence-associated genes have been identified, our findings align with previous studies on serum resistance in *E. coli*, particularly those linked to the prevalence of *traT* in clinical strains [37,38]. These findings have significant implications in managing and preventing sepsis, particularly in patients with comorbidities [55].

The diversity of virulence determinants influences the clinical presentation and treatment outcomes. Strains harbouring multiple virulence genes exhibit heightened pathogenic potential, leading to severe infections and poor patient prognosis. Specific factors such as adhesins and biofilm-associated genes complicate antimicrobial therapy by enhancing bacterial persistence and resistance. Understanding this diversity is crucial to develop targeted treatment strategies and improve sepsis outcomes. Functional studies linking virulence gene expression to clinical phenotypes are critical for advancing precision medicine approaches to sepsis management. In addition, incorporating genomic insights into clinical workflows can support predictive models of infection severity and optimize therapeutic interventions.

### SNP analysis

SNPs catalogued from each genome were consolidated into a unified SNP panel, reporting positions, allelic variations, genic/intergenic statuses and annotations. Sequencing results were compared against those of the reference strain (*E. coli* K-12, NC_000913.3), confirming the presence of significant alleles. Our benchmark analysis revealed that while many SNP callers identified false positives, SnpEff demonstrated high accuracy, owing to its optimized filtering parameters. This approach enabled sensitive detection of SNP, particularly in key AMR and VF-associated genes. The identified SNPs highlight critical genetic adaptations in *E. coli* genomes that contribute to AMR and virulence. SNPs in *β*-lactamase and efflux pump genes may enhance resistance through structural or regulatory changes, while variations in virulence genes encoding adhesins, toxins and protectins could influence host–pathogen interactions and infection severity [43]. These findings emphasize the evolutionary mechanisms driving resistance and pathogenicity and offer valuable insights into therapeutic interventions. Further functional studies are required to elucidate the precise roles of these SNPs in modulating bacterial phenotypes.

### AMR in *E. coli*

All *E. coli* genomes exhibited resistance to *β*-lactam antibiotics, including cephalosporins, cephamycins and penicillins, by hydrolysing the *β*-lactam ring through ESBLs (e.g. *blaCTX-M-15* and *blaTEM-1B*). These enzymes resist third- and fourth-generation cephalosporins such as cefotaxime, ceftriaxone, ceftazidime and monobactams such as aztreonam. Additionally, ESBLs mediate MDR by hydrolysing carbapenems and other *β*-lactams, severely limiting treatment options. Interestingly, the VITEK 2 Compact System failed to detect these ESBL genes, highlighting its limitations in clinical settings.

### Unrecognized genes with AMR potential

Among the 88 identified AMR-associated genes, several previously unrecognized candidates were linked to resistance phenotypes. Notable examples include *tsh*, *pic*, *mecA*, *lmo*, *cadC*, *blaOKP-A/B*, *cnf1*, *blaOXY-1/4/5*, *arsR-K-12*, *vanS-A/E*, *arsC_gluta*, *blaOXA-12/51/184* and *CMY2-MIR-ACT-EC*. These genes and others, such as *rho-1/2*, *aacA-STR-10*, *vanS-N*, *blaPEDO-2*, *sat* and *vanG-Cd,* contribute to the AMR landscape. This study also identified unique gene variants, including *vanTm-L*, *ariR*, *ble-MBL*, *blaOXA-23/62/134* and *blaPDC_var*. Although these genes have not yet been well characterized, their presence in MDR *E. coli* isolates suggests that genes contribute significantly to resistance mechanisms. Genes such as *blaOXA* encode *β*-lactamases that hydrolyse *β*-lactam antibiotics, while others, like *tsh* and *pic*, may influence biofilm formation and immune evasion, indirectly promoting resistance. Regulatory genes such as *cadC* may modulate resistance pathways through transcriptional regulation.

Further research is required to determine the precise roles of these genes in resistance and pathogenesis. Experimental studies and computational modelling can elucidate their interactions with known resistance pathways and identify potential targets for therapeutic interventions. Expanding AMR databases to incorporate these novel determinants is essential for improving diagnostic accuracy and treatment strategies.

### AMR in *Enterobacteriaceae* infections among ICU patients

Infections caused by *Enterobacteriaceae*, including *E. coli* and *K. pneumoniae*, are of significant concern in ICU patients with MDR sepsis. Studies have reported an incidence rate of ~51% among ICU patients, with infection incidence densities ranging from 13 to 20.3 episodes per 1,000 patient-days [39]. A study conducted between June 2009 and December 2013 reported a 14.9% mortality rate in patients with *Enterobacteriaceae* infections, sepsis, severe sepsis or septic shock. Mortality rates increased significantly with sepsis severity: 3.5%, 9.9% and 28.6% for patients with sepsis, severe sepsis and septic shock, respectively. The study further found that, while the time to antimicrobial therapy was not significantly associated with mortality, prolonged ICU and hospital stays were significantly correlated with higher sepsis severity, ultimately increasing mortality rates in patients with sepsis [56].

Another study revealed that 48% of patients infected with *Enterobacteriaceae* developed recurrent infections within a 12-month follow-up period. Over half of these recurrent infections were caused by the same bacterial species and occurred at the same culture site. MDR Gram-negative bacterium was identified as an independent predictor of mortality after discharge. The study also found that the risk of recurrent infection was the highest within the first 3 months post-discharge, emphasizing that this period is critical for monitoring and intervention [57]. These findings highlight the importance of timely and accurate prognosis and management strategies for ICU patients with sepsis and MDR *Enterobacteriaceae* infections. Although our hospital-based study focused on patients with sepsis and associated comorbidities, managing bloodstream infections caused by Gram-negative Bacillary Bloodstream Infection (GN-BSI) bacteria in severe cases remains a continuous issue. Notably, our study did not directly address the mechanisms of host–genome interactions.

### Challenges in managing GN-BSI and AMR

This study focused on patients with sepsis and bloodstream infections caused by Gram-negative Bacillary Bloodstream Infection (GN-BSI), particularly MDR strains. Persistent GN-BSI (positive blood culture) was associated with a significantly higher mortality than non-persistent GN-BSI (negative blood culture). These findings emphasize the importance of understanding host–genome interactions to identify genes driving AMR and VF expression. Such insights could help mitigate the effects of inadequate source control and inappropriate antibiotic therapy. Further investigations are required to elucidate these mechanisms and their impact on resistance. This study aligns with existing studies and underscores the need for policy interventions, enhanced infection control measures and increased investments in AMR research. This study’s extensive bioinformatic and machine learning approaches provide actionable insights into AMR profiles, enabling more precise treatment strategies. Our bioinformatic analyses yielded accurate comparisons of MDR and susceptible *E. coli* strains, with all 18 isolates demonstrating resistance to multiple antibiotics. Future studies will extend these methodologies to analyse the AMR and VF genes in 18 *E. coli* isolates from patients with sepsis.

### Key insights into AMR and pathogenicity

This study highlights the challenges of AMR in *E. coli* infections, particularly in sepsis. Diverse *E. coli* strains have been identified, including the ESBL-producing ST131 strain, which is commonly linked to UTIs. Bioinformatic tools were employed to predict virulence- and pathogenicity-associated genes, while Circos visualization was used for structural analysis of the *E. coli* pathogenicity island. These analyses provide significant insights into AMR determinants such as ESBL genes and reveal the structural characteristics of *E. coli* genomes, thus providing a foundation for targeted treatment strategies to improve patient outcomes.

### Conclusion and future directions

This research underscores the urgent need for tailored sepsis management strategies, especially in regions such as India, where antibiotic misuse exacerbates AMR. These findings emphasize the importance of integrating genomic approaches to treat MDR pathogens effectively. By identifying specific AMR determinants (e.g. blaCTX-M-15) and VFs (e.g. adhesins and toxins), our findings offer a framework for tailoring antibiotic therapies and predicting infection severity. The detection of ESBL-producing strains like ST131 can guide the use of carbapenems or combination therapies while avoiding ineffective *β*-lactams, thereby optimizing treatment outcomes. Integrating genomic insights into clinical workflows could enhance diagnostic precision and reduce the burden of MDR sepsis in hospital settings. Future research will extend these methods to explore AMR and virulence mechanisms in other critical pathogens, such as *K. pneumoniae*, and refine bioinformatic tools for more precise interventions.

## Abbreviations

AKI: acute kidney infection
AMR: antimicrobial resistance
ANI: average nt identity
AST: antibiotic susceptibility testing
AUC: area under the curve
CDS: coding sequence
COG: Clusters of Orthologous Groups
ESBL: extended-spectrum *β*-lactamase
gDNA: genomic DNA
GO: Gene Ontology
ICU: intensive care unit
KEGG: Kyoto Encyclopedia of Genes and Genomes
LR: logistic regression
L-SVM: linear support vector machine
MDR: multidrug resistance
MODS: multiple organ dysfunction syndrome
NCBI: National Center for Biotechnology Information
ncRNA: noncoding RNA
RBF-SVM: radial basis function support vector machine
SMOTE: synthetic minority oversampling technique
SOFA: Sequential Organ Failure Assessment
ST: sequence type
UTI: urinary tract infection
VF: virulence factor
WGS: whole-genome sequencing
WHO: World Health Organization
